# Influence of dietary oils rich in omega-6 or omega-3 fatty acids on rumen microbiome of dairy cows

**DOI:** 10.1093/tas/txad074

**Published:** 2023-07-01

**Authors:** Nathaly Cancino-Padilla, Felipe Gajardo, André Luis Alves Neves, Ahmed Eid Kholif, Marcello Mele, Sharon A Huws, Juan J Loor, Jaime Romero, Einar Vargas-Bello-Pérez

**Affiliations:** Pontificia Universidad Católica de Chile, Departamento de Ciencias Animales, Facultad de Agronomía e Ingeniería Forestal, Santiago 4860, Chile; Instituto de Investigaciones Agropecuarias, INIA Carillanca, Temuco, Chile; Universidad de Chile, Instituto de Nutrición y Tecnología de los Alimentos (INTA), El Líbano 5524, Macul, Santiago, Chile; Department of Veterinary and Animal Sciences, University of Co-penhagen, Grønnegårdsvej 3, 1870 Frederiksberg C, Denmark; Dairy Science Department, National Research Centre, Giza, Egypt; Dipartimento di Scienze Agrarie, Alimentari e Agro-ambientali, Università di Pisa, 56124 Pisa, Italy; Queen’s University of Belfast, Institute for Global Food Security, School of Biological Sciences, Belfast, BT9 7BL, UK; Department of Animal Sciences and Division of Nutritional Sciences, University of Illinois, Mammalian NutriPhysioGenomics, Urbana 61801, USA; Universidad de Chile, Instituto de Nutrición y Tecnología de los Alimentos (INTA), El Líbano 5524, Macul, Santiago, Chile; Pontificia Universidad Católica de Chile, Departamento de Ciencias Animales, Facultad de Agronomía e Ingeniería Forestal, Santiago 4860, Chile; Department of Animal Sciences, School of Agriculture, Policy and Development, University of Reading, Reading RG6 6EU, UK

**Keywords:** fatty acids, fish oil, oilseeds, milk, rumen, vaccenic acid

## Abstract

The objective of this study was to compare the effect of supplementing dairy cow diets with contrasting sources of omega-6 (soybean oil) and omega-3 (fish oil) PUFA on rumen microbiome. For 63 d, 15 mid-lactating cows were fed a control diet (*n* = 5 cows; no fat supplement) or control diet supplemented with 2.9% dry matter (DM) of either soybean oil (**SO**; *n* = 5 cows) or fish oil (**FO**; *n* = 5 cows). Ruminal contents were collected on days 0, 21, 42, and 63 for 16S rRNA gene sequencing. Beta diversity and Shannon, Simpson and Chao1 diversity indices were not affected by dietary treatments. In terms of core microbiome, *Succiniclasticum*, *Prevotella*, *Rikenellaceae*_RC9_gut_group, and NK4A214_group were the most prevalent taxa regardless of treatments. *Bifidobacterium* was absent in SO diet, *Acetitomaculum* was absent in FO, and *Sharpea* was only detected in SO. Overall, results showed that at 2.9% DM supplementation of either SO or FO over 63 days in dairy cow diets does not cause major impact on bacterial community composition and thus is recommended as feeding practice.

## Teaser

This study showed mild effects of dietary lipids on rumen microbiome in cows. Minor effects can occur when cows are fed with 2.9% DMinclusion of dietary polyunsaturated fatty acids.

## Introduction

Dietary polyunsaturated fatty acids (**PUFA**) in dairy cow result in different milk FAs, many of which have positive effects on human health (Nguyen et al., 2019). Examples of such FAs are vaccenic (C18:1trans11) and rumenic (C18:2 cis9, trans11) acids (Gómez-Cortés et al., 2018). To increase the concentration of those FAs, several nutritional strategies have been carried out and addition of vegetables (i.e., by-products from oilseeds) (Morsy et al., 2015; Kholif et al., 2018; Plata-Pérez et al., 2022) and marine (i.e., salmon oil) (Kairenius et al., 2015) lipid sources have been effective for improving milk FA profile in terms of human health properties. When cows are fed with these dietary lipid sources, changes at rumen (Cancino-Padilla et al., 2021), adipose tissue (Vargas-Bello-Pérez et al., 2019a) and mammary gland (Vargas-Bello-Pérez et al., 2019c) levels occur and understanding how dietary FAs are metabolized is important to improve milk fat quality linked to human health (Vargas-Bello-Pérez et al., 2020). The extent of the changes caused by dietary FA will depend on many factors, including forage to concentrate ratio, inclusion level of the dietary lipid source and even the individual FA structure, such as chain length, degree of saturation, and orientation of FA double bonds (Vargas-Bello-Pérez et al., 2019b).

Ruminal microbiome is one of the most interesting and complex ecosystems in nature, being composed on bacteria, protozoa, fungi, archaea, and viruses (mainly phages) that have a strong influence on animal production (John Wallace et al., 2019) due to their involvement in the degradation of plant cell walls and their conversion of glucose precursors to volatile fatty acids, such as propionic acid (Chen et al., 2020). The ruminal microbiome also has an important role in FA metabolism of dietary fats, particularly with respect to rumen biohydrogenation (Vargas-Bello-Pérez et al., 2016), which is a process whereby double bonds of dietary fats are removed by rumen bacteria as they are toxic to them, and by doing so, microorganisms survive while saturated FA contents are increased in milk or meat (Bionaz et al., 2020).

Previously, unprotected soybean oil and salmon oil were fed to lactating dairy cows, and although both oils are considered PUFA sources, their FA structure (i.e., number and location of double bonds) led to differential effects on the expression of genes related to lipid metabolism (related FA import into cells, FA synthesis and desaturation, acetate and FA activation and intra-cellular transport, triacylglycerol synthesis, lipid droplet formation and regulation of transcription) in subcutaneous adipose tissue (Vargas-Bello-Pérez et al., 2019a), and mammary gland (Vargas-Bello-Pérez et al., 2019c). However, it remains unknown as to how soybean oil or salmon oil affect rumen microbiome. Although both oils are considered polyunsaturated FA sources their effects on microbiome are expected to be different due to their FA chemical configuration and this has not been reported. Therefore, the objective of this study was to compare the effect of supplementing dairy cow diets with contrasting sources of omega-6 and omega-3 PUFA on the rumen microbiome. The hypothesis of this study was that rumen microbiome changes will depend on the degree of FA saturation and number of double bonds, and this should be more pronounced with marine oil. Also, the influence of the dietary components is not just associated with one taxon but could be associated with several groups or changes in beta diversity. For that, soybean oil (**SO**) was used as a source of C18:2 cis n-6 (50 g/100 g), and fish oil (**FO**) was used as a source of C20:5 n-3 (16 g/100 g) and C22:6 n-3 (8 g/100 g) over a relatively long period of supplementation (63 d). The supplementation level of these oils was 2.9% dry matter as this level has been reported to modulate milk fatty acid profile (Vargas-Bello-Pérez et al., 2019b) without negative effects on animal’s metabolism (Vargas-Bello-Pérez et al., 2019a, 2019c) or production parameters.

## Materials and Methods

### Animal Conditions and Experimental Design

The Animal Care Committee of the Pontificia Universidad Católica de Chile approved all the experimental procedures (project ID 160809002), in accordance with their animal care, animal welfare, and procedures guidelines, performed at the Estación Experimental Pirque of the Fundación AgroUC (33°38ʹ28″S, 70°34ʹ27″W). Animals were housed in individual stalls (2.4 × 6 m) and with ad libitum access to water.

### Dietary Treatments

All cows received an isocaloric basal restricted diet (NEL = 1.6 Mcal/kg DM) containing 65% forage (corn silage, fresh alfalfa, and alfalfa hay) and 35% concentrate (malt distillers, corn grain, wheat bran, soybean grain, and rapeseed meal) to satisfy the nutritional requirements of a 650-kg dairy cow in mid-lactation consuming 26.5 kg DM daily (Vargas-Bello-Pérez et al., 2019b). Treatments included a control basal diet with no added lipid (*n* = 5 cows), and a basal diet containing either SO (*n* = 5 cows; unprotected soybean oil; 2.9% DM) or fish oil (*n* = 5 cows; unprotected salmon oil; 2.9% DM). Animals were dietary treatments over 63 and on day 0, all animals received the control diet. Oils were mixed and supplied manually into the daily ration for each cow. Details on diets, oils, and animals are reported in a companion paper (Vargas-Bello-Pérez et al., 2019b).

### Ruminal Samples Analysis

Individual rumen samples were taken on days 0, 21, 42, and 63 using a transesophageal scoop (FLORA Rumen Scoop, Geishauser, Wittibreut, Germany) after morning milking and before feeding. Approximately 15 mL of the liquid fraction containing particulate matter (particles up to 10 mm) was removed from the rumen following Geishauser et al. (2012) protocol. All technical details on the rumen scoop mechanism, and maintenance while sampling and between samplings have been reported previously (Larsen et al., 2020).

### Ruminal Metataxonomic Analysis

Frozen (−80 °C) rumen fluid samples were thawed on ice and then homogenized with vortex and 250 mg was weighed in 1.5 mL Eppendorf tubes. Then, 150 μL of phosphate-buffered saline (PBS) was added to each sample to perform cell lysis with lysozyme incubation at 37 °C for 60 min, as a pre-treatment for DNA extraction. Consequently, DNA was extracted using the UltraClean Fecal DNA Isolation Kit (MO BIO Laboratories, Carlsbad, CA, USA) according to the manufacturer’s protocol, which involved physical and chemical disruption of cell membranes. Ruminal samples from five animals/treatment were used for sequencing.

The extracted DNA underwent 16S rRNA gene amplification using the bacterial-specific primers 515F 5ʹ-GTGCCAGCMGCCGCGGTAA-3ʹ and 806R 5ʹ-GGACTACHVGGGTWTCTAAT-3ʹ (Caporaso et al., 2011), to amplify the V3 to V4 regions of the 16S rRNA gene (Lokesh and Kiron, 2016). Polymerase chain reaction was performed using the following conditions: an initial denaturing cycle of 5 min at 94 °C, followed by 35 cycles of 30 s at 94 °C, annealing at 56 °C for 30 s, and an elongation at 68 °C for 45 s. After 16S rDNA V4 region amplification, PCR products were purified through QIAquick PCR Purification kit (Qiagen, Valencia, CA). Subsequently, the purified products were quantified fluorometrically using the High Sensitivity (HS) kit on the Qubit Fluorometer 3.0 (Invitrogen Co., Carlsbad, CA, USA).

DNA sequencing was performed by CD Genomics (New York, NY, USA) using the Illumina MiSeq 2 × 300 platform. The 16S rRNA gene amplicon sequences were quality checked with FASTQC and analyzed using DADA2 and Phyloseq R package version 3.5.1. The quality threshold used for quality filtering of reads was over 28, for forward and reverse reads. Sequences were trimmed to 270 (forward) and 220 bp (reverse). The paired-end Illumina reads were assembled into amplicon sequence variants(**ASV)** using the DADA2 pipeline (Supplementary Table S1). Taxonomy assignation was performed using the Silva training dataset version 138, and sequences corresponding to Eukaryota, Crenarchaeota, and Euryarchaeota at the phylum level were removed from the analysis.

### Statistical Analysis

Alpha and beta diversity were estimated from the complete bacterial amplicon sequence variant (ASV) table. Alpha (within-sample diversity) and beta diversity (between-sample diversity) measures for samples, grouped by dietary treatments and experimental periods, were analyzed using the phyloseq package in R (McMurdie and Holmes, 2013). Microbial diversity was determined using the Shannon Index (combines richness or the total number of taxa and evenness, the relative abundance of each taxon), dominance was presented using the Simpson index; and richness of samples was calculated based on the Chao1 index and observed species. Beta diversity was calculated using the UniFrac metric and principal coordinates analyses (**PCoAs**) using both weighted (quantitative) and unweighted (qualitative) Unifrac distances, to highlight clusters of similar groups of samples depending on the diet supplementation. In addition, ANOSIM was used to elucidate the differences in microbial communities among the three different treatments.

Differential analysis of the taxonomic profile and metabolic pathways. First, we used CowPI (Wilkinson et al., 2018) to infer enzymatic functions from 16S rDNA amplicon sequencing data from rumen samples. As a result, we obtained a table with several Kyoto Encyclopedia of Genes and Genomes (KEGG) categories associated with abundance estimates in each sample (Table 1). Next, we used the LEfse method, as implemented in the Microbiome Analyst webpage, to detect differences in abundance, associated with three different diets: Control, FO, and SO. We were able to obtain 28 enzymatic functions with a false-discovery rate (**FDR**) lower than 1%, on which we performed a hierarchical clustering considering the abundance estimates for samples of each diet. Thus, we identified five clusters with characteristic patterns of abundance (height tree parameter set to 25). This complete analysis, together with a heatmap visualization of the results was done using a custom script implementing the hclust and cutree functions from the stats package in R.

**Table 1.** KEGG categories associated with abundance estimates from control, soybean oil and fish oil

KEGG	*P*-values	FDR	Control	Fish oil	Soybean oil	LDA score	Gene	Description
K01795	0.0036719	0.14847	39.769	4.541	41.148	108	algG	mannuronan 5-epimerase [EC:5.1.3.37]
K02585	0.003031	0.14175	15.603	10.843	15.003	0.0927	nifB	nitrogen fixation protein NifB
K01225	0.0011172	0.07093	12.141	0.71643	11.631	0.0965	CBH1	cellulose 1,4-beta-cellobiosidase [EC:3.2.1.91]
K13571	0.0036996	0.14847	0.09090	0.05233	0.26015	0.0429	pafA	proteasome accessory factor A [EC:6.3.1.19]
K13527	0.0033559	0.14733	0.09090	0.05204	0.26015	43	mpa	proteasome-associated ATPase
K01354	0.0041278	0.15834	0.20378	0.13014	0.36408	48	ptrB	oligopeptidase B [EC:3.4.21.83]
K10559	0.0052417	0.18641	0.12817	0.28939	0.21358	0.0337	rhaS	rhamnose transport system substrate-binding protein

KEGG: accession number for the KEGG database; *P*-values: statistical significance as reported by LEfSe; FDR: false discovery rate as reported by LEfSe; control, fish oil, and soybean oil columns contains the abundance of every marker on each treatment; LDA score: linear discriminant analysis score, is a metric of the relative contribution of each marker to the difference between treatments; Gene: the gene symbol of the identified marker; description: the name of the corresponding marker.

## Results

### Production Traits

Milk yield, milk composition, and milk fatty profiles have been reported previously (Vargas-Bello-Pérez et al., 2019b). Dry matter intake (26.5 kg of dry matter per day), milk yield (43 kg/day), milk fat (1.52 kg/day), and milk protein (1.50 kg/day) were similar between treatments. Saturated fatty acids were reduced with SO and FO. C18:2 cis n-6 was increased with SO, while C18:2 cis-9, trans-11, C20:3 n-3, C20:3 n-6, C20:5 n-3, and C22:6 n-3 were greater with FO.

### Ruminal Bacterial Diversity Analysis

Sequencing the V4 region of the bacterial 16S rRNA gene produced 4,488,309 reads (joined R1 to R2 paired-end reads). After data filtering, quality control, and chimera removal, a total of 1,552,438 V4 16S rRNA sequence reads from the 60 samples remained, and a mean of 25,873 reads for each sample (minimum, 9,776; maximum, 39,820). Four samples for each treatment and period (60 samples) were used for sequencing. A total of 7,099 ASV were obtained after analysis with Phyloseq from the bacterial 16S rRNA gene sequencing, which was taxonomically grouped from the phylum to genus level (phylum, class, order, family, and genus).

**Figure 1.**
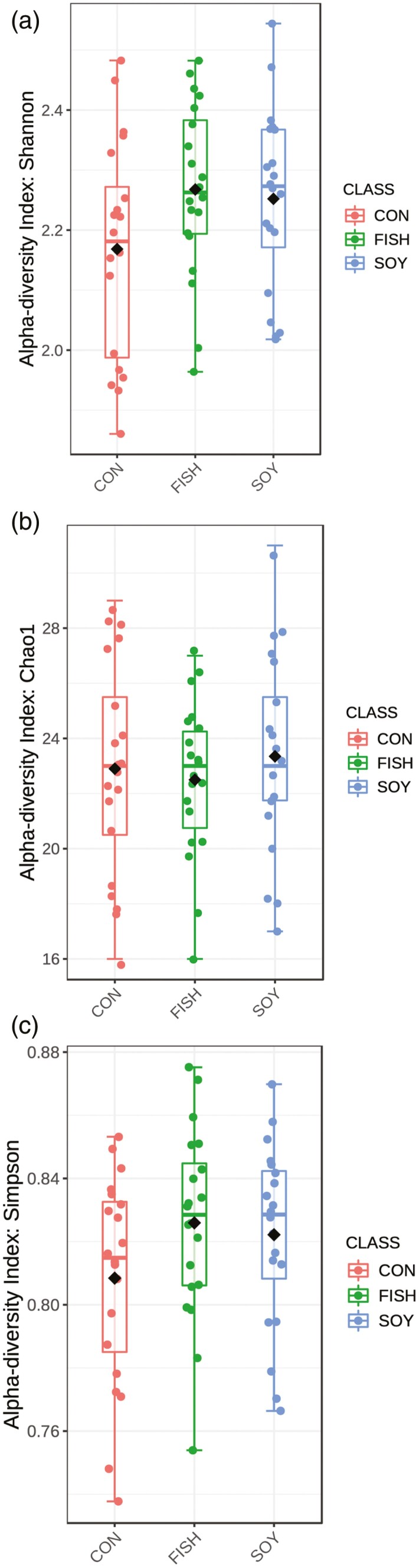
Ruminal microbial richness and diversity with lipid supplementation throughout the experimental periods. Bacterial diversity estimated by (a) Shannon Index and (b) Simpson Index, and bacterial richness estimated by the (c) Chao1 value. CON, no fat supplement; SOY, supplemented with 2.9% DM soybean oil; FISH, supplemented with 2.9% DM fish oil. Supplementation periods of 0, 21, 42, and 63 d.

**Figure 2.**
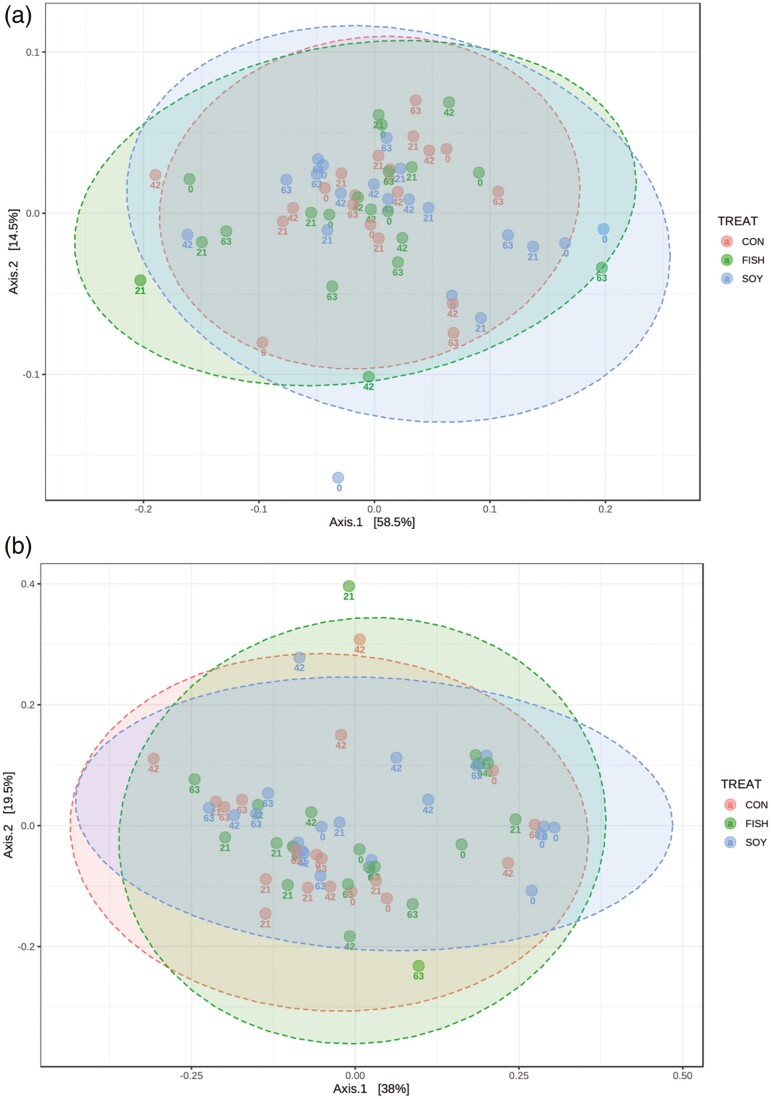
Principal coordinate analysis (PCoA) of bacterial community structures of the ruminal microbiota in the control (CON, red points), FISH (green points) and SOY (blue points), constructed using the (a) weighted UniFrac and (b) unweighted Unifrac method. CON, no fat supplement; SOY, supplemented with 2.9% DM soybean oil; FISH, supplemented with 2.9% DM fish oil. Supplementation periods of 0, 21, 42, and 63 d.

Additionally, for the diversity analyses, we performed a subsampling by read coverage considering 10,682 reads per library. Alpha diversity was not altered by diet. Shannon (Figure 1a; *P*-value = 0.11), Chao1 (Figure 1b; *P*-value = 0.73), and Simpson (Figure 1c; *P*-value = 0.17) diversity indices were not significantly different between each dietary treatment. Beta diversity between the samples at four different time points during lipid supplementation was evaluated. In both the weighted (Figure 2a) and unweighted (Figure 2b) UniFrac distances, the closer positions of the samples in the PCoA, indicated similar microbial composition. Nevertheless, permutational multivariate analysis of variance analysis on the unweighted UniFrac distances revealed significant differences only between control and fish oil treatments (*P*-value = 0.009), contrary to the soybean oil treatment (*P*-value = 0.2). On the other hand, when using the same methodology for comparing between days of supplementation, we did not find significant differences on any treatment (21 vs. 42 d: *P*-value = 0.119; 21 vs. 63 d: *P*-value = 0.051).

**Figure 3.**
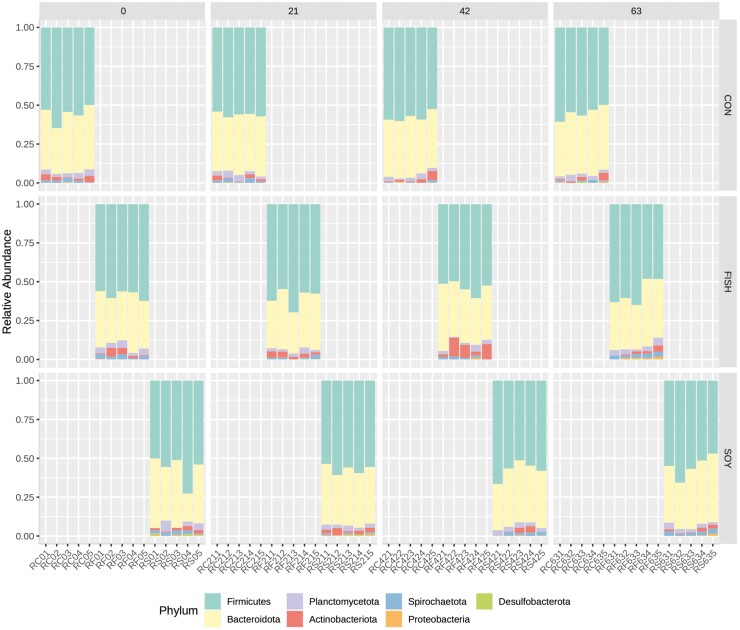
Microbiota composition at phylum level. Microbiota during the assay at days 0, 21, 42, and 63 and cows fed different diets: control (CON), soybean oil (SOY), and fish oil (FISH). Relative abundance is showed in the scale 0 to 1.

At the phylum level, seven phyla were identified in the ruminal samples irrespective of diet (Figure 3), with phyla Firmicutes (56.9%), and Bacteroidetes (35.9%), being the most abundant followed by Planctomicetota (2.8), Actinobacteria (2.3%), Spirochaetota (1.5%), Proteobacterias (0.4%), and Desulfobacterota (0.2%) (Figure 3). Seventeen bacterial families were identified within rumen samples. The main families were Acidaminococcaceae (31.3%) and Prevotellaceae (19.7%), followed by Oscillospiraceae (8.0%), Rikenellaceae (7.5%), Lachnospiraceae (4.9), Christensenellaceae (4.8%), Anaerovoracaceae (4.5%), and others representing lower proportions.

**Figure 4.**
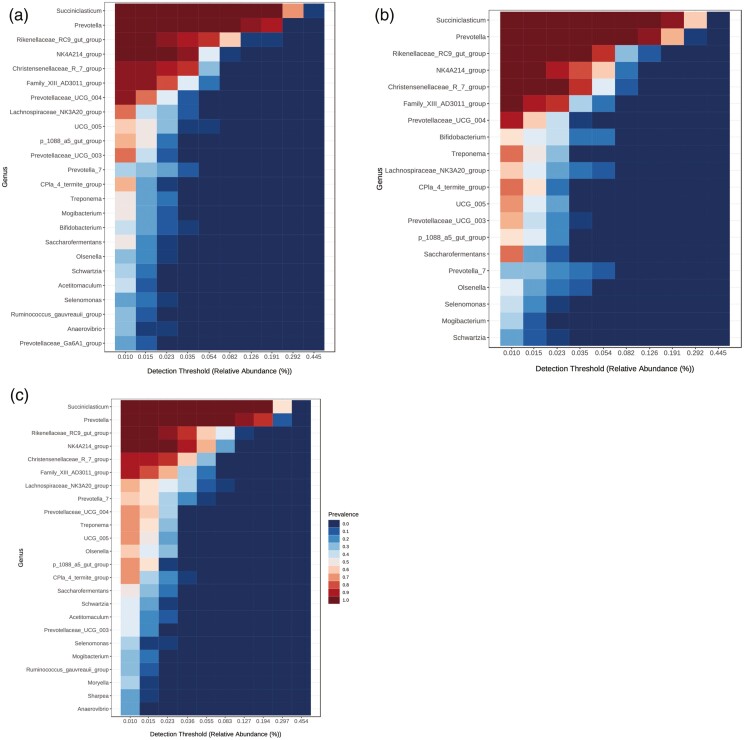
Core microbiome in each diet. Bacterial diversity estimated by (a) Control, (b) Fish oil (c) Soybean oil. CON, no fat supplement; SOY, supplemented with 2.9% DM soybean oil; FISH, supplemented with 2.9% DM fish oil.

Relative abundances at genus level shown in Supplementary Figure S1. *Succiniclasticum* (Firmicutes/Acidaminococcaceae) and *Prevotella* (Bacteroidetes/Prevotellaceae) were dominant, with mean relative abundance of 31.3% and 22.8%, respectively. They were followed by *Rikenellaceae*_RC9_gut_group (7.5%), *NK4A214_group* (6.2%), *Christensenellaceae*_R_7_group (4.8%), *Family_XIII_AD3011_group* (3.6%), and other representing lower proportions (Figure 4). In terms of the core microbiome, components at the genus level were illustrated in Figure 4. *Succiniclasticum*, *Prevotella*, Rikenellaceae_RC9_gut_group, and *NK4A214_*group were the most prevalent taxa in the three diet groups. However, this analysis showed some components differentially distributed, such *Bifidobacterium* that is absent in SO, *Acetitomaculum* absent in FO, and *Sharpea*, only present in SO.

### Linear Discriminant Analysis Effect Size and Sparse Correlations for Compositional Data (SparCC)

**Figure 5.**
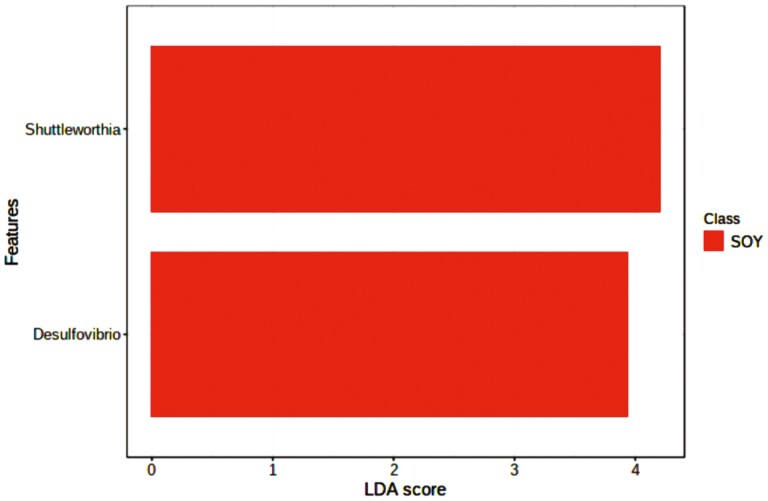
Linear discriminate analysis (LDA) effect size (LEfSe). This shows the taxa differentially associated with one of the three diets.

The linear discriminant analysis (LDA) effect size (LEfSe) was used to identify possible discriminating taxa among diet groups. Only 2 distinguishing taxa were detected among the groups of Control, FO and SO showing LDA score > 3. *Shuttleworthia* and *Desulfovibrio* were associated with SO (Figure 5).

**Figure 6.**
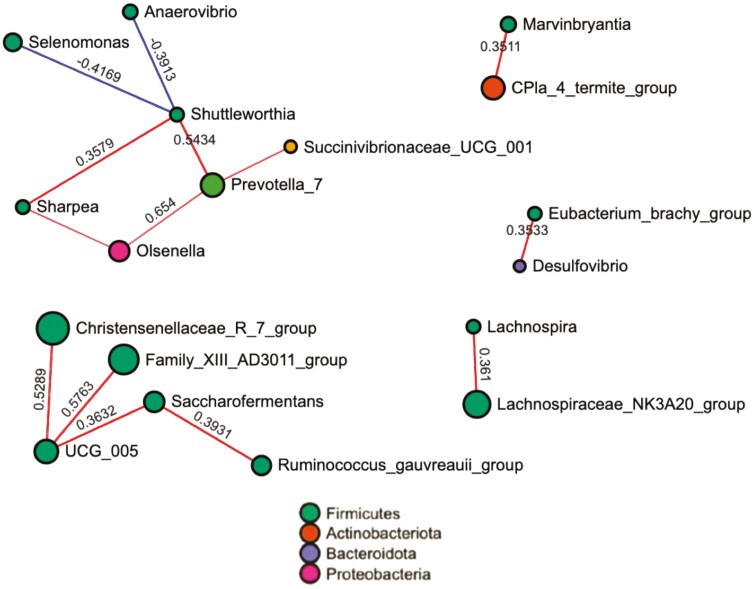
Correlations among the taxa using SparCC. Each node represents a taxon, the color depends on the phylum. Node size represents the number of connections to that taxon. Red lines represent positive correlations, blue lines negative correlations.

In terms of correlations, the tool SparCC was used to illustrate the relationships among the taxa within the rumen (Figure 6). It was observed that Firmicutes had positive correlations in most of the nodes. Interestingly, the correlations were positive between Family XIII_AD3011_group and UCG_005 and *Saccharofementans*, whilst *Ruminococcus_gauvreauii*_group had negative correlations with *Desulfovibrio* and this with *Bifidobacterium*. In the other group, *Prevotella*_7 had positive correlations with *Shuttleworthia*, *Olsenella*, and *Lach-nospiraceae*_NK3A20_group.

### Predicted Metabolic Pathways

Predicted functional features of the rumen bacterial community obtained using CowPi showed 500 gene families identified in ruminal samples. Those functions were discriminated by LEfSe and clustered in a heatmap (Supplementary Figure S2). Some differences in the predicted pathways can be observed. Seven functions showed significant differences among the dietary treatments (Table 1). Those functions were related to protein metabolisms, sugar metabolism, and nitrogen fixation (Table 1).

## Discussion

Determining the effects of diet on rumen microbiome is important as it is closely connected with animal’s productivity and health (O’Hara et al., 2020; Liu et al., 2021) and thus, to avoid detrimental effects, any dietary supplement should be tested. In this regard, the present study, aimed at determining the effects of adding SO or FO on rumen bacteria composition.

In this study, ruminal diversity was not affected by treatments suggesting that supplemental fat from vegetable or marine origins at 2.9% DM was not detrimental to the maintenance of a stable ruminal bacterial community. Similarly, when lactating cows were fed diets with 3% DM soybean oil or hydrogenated vegetable oil, no changes were noted in ruminal alpha diversity or Shannon, Chao1, and Simpson diversity indices. Conversely, Bayat et al. (2018) proposed that lipid supplements (50 g/kg of dry matter) alter the diversity of ruminal microbial communities and relative abundance of some common taxa, as opposed to inducing a global response. Huws et al. (2015) reported that the number of bacterial populations (species richness) and their distribution (diversity) changed in response to different oil supplements (30 g/kg of dry matter), possibly due to level of supplementation fed in those studies. In this study, the level of oil inclusion was slightly lower compared to previous studies. Normally, the recommended dietary fat levels for lactating cows are not above 5% DM (Bionaz et al., 2020). Interestingly, in other types of ecosystems, increases in biodiversity are associated with greater success and resilience of the ecosystem (Cardinale et al., 2002; Zak et al., 2003), but not for the ruminal environment.

The phyla Firmicutes and Bacteroidetes were the most preponderant irrespective of treatments, and normally those are the dominant phyla in rumen microbiome (Henderson et al., 2015). The ratio of Firmicutes to Bacteroidetes has been correlated with milk fat yield (Jami et al., 2014). In this study, milk fat yield was similar, which also agrees with findings from these phyla abundance.

Acidaminococcaceae was the preponderant family, and it is recognized for its ability to digest complex fiber, pectin, and nitrogen-containing substrates, in addition, this family can use amino acids as their sole source of energy for their growth and development (Ryazanov et al., 2021). In this study, Prevotellaceae was the second most abundant family and together with its genera have been associated with the breakdown of dietary proteins and carbohydrates in feed (Zhang et al., 2021). These findings suggest that at family level, dietary oils from vegetable or marine origin do not interfere with protein metabolism which is very important in the context of dairy production as it may affect energy supply, milk yield, and milk components.

*Succiniclasticum* and *Prevotella* were the dominant genera. *Succiniclasticum* has synergy with cellulolytic bacteria and helps to convert succinate to propionate as a solid energy-yielding mechanism (Du et al., 2022). *Prevotella* has been reported to be greatly involved in N usage and has been positively correlated with urinary N excretion (Gomes Carvalho Alves et al., 2021). In the current study, both genera were not affected showing that treatments did not disrupt glucose precursors and N-related by-products escaping from the rumen.

Regarding the core microbiome, Bifidobacterium was absent in the SO, and this is considered a probiotic that has been found to be related to increases in rumenic acid production (Park et al., 2011). This FA was higher with FO (1.75 g/100 g), intermediate for SO (1.18 g/100 g), and lowest in the control (0.50 g/100 g). Full milk fatty acid profiles have been reported previously (Vargas-Bello-pérez et al., 2019b). *Acetitomaculum* was absent in FO, and abundance of this microorganism has been related to increases in dietary energy, where feeding high-concentrate diets enhances monosaccharide availability in the rumen to produce acetate which provides substrate for *Acetitomaculum* (Wang et al., 2019). *Sharpea* was only present in SO and ruminal abundance of this genus has been positively correlated with trans-10 intermediates and has been reported to be alternative biohydrogenator of linoleic and linolenic acids (Dewanckele et al., 2019). This may explain why SO (rich in linoleic acid) increased the presence of this microorganism. Data from the core microbiome revealed minor but specific effects on different aspects such as the role that SO had on the formation of different ruminal biohydrogenation by-products resulting in secretion of rumenic acid in milk. This FA has been reported to have positive effects on human health (Gómez-Cortés et al., 2018; Nguyen et al., 2019).

*Shuttleworthia* and *Desulfovibrio* were related to SO and exhibited positive correlations with *Shuttleworthia*, *Olsenella*, and *Lachnospiraceae*_NK3A20_group. *Desulfovibrio* negatively correlated with *Bifidobacterium*. *Shuttleworthia* are saccharolytic bacteria that produce acetate, butyrate, and lactate as end-products of glucose fermentation while *Desulfovibrio* are known as the group of sulfate-reducing bacteria (Xiao et al., 2016). These results are somehow related to the abovementioned effects of SO on different taxa. In addition, data from predicted metabolic pathways reinforce our findings on productive traits and rumen microbiome analysis. Compared to control and FO, SO has shown minor effects on N metabolism and energy utilization in the rumen.

## Conclusions

Although soybean oil and fish oil were characterized by differences in their individual FA (i.e., linoleic acid, and eicosapentaenoic acid or docosahexaenoic acid) profiles, ruminal microbiome changes due to feeding these oils were minor, a response also observed in our companion papers at the subcutaneous adipose tissue (Vargas-Bello-Pérez et al., 2019a) and mammary gland (Vargas-Bello-Pérez et al., 2019c) levels. Overall, this study brought insights into the changes at the ruminal level suggesting that soybean oil and fish oil can be included in mid-lactating cow diets without detrimental effects on global bacteria composition and the quality of the milk. Future efforts should be directed to understanding the ruminal metabolome, as the identification of specific metabolites from lipid metabolism (Zeng et al., 2019) can help improve feeding management for obtaining healthier milk fat. Adding either soybean oil or fish oil at 2.9% DM to mid-lactating cow diets does not affect the ruminal microbiome. These feedstuffs can be used over relatively long periods of time without significant changes on ruminal microbiome diversity and metataxonomy.

## Supplementary Material

txad074_suppl_Supplementary_Figures

## Data Availability

The raw data supporting the conclusions of this article will be made available by the authors, without undue reservation.
