# Supplementary material for: Influence of dietary oils rich in omega-6 or omega-3 fatty acids on rumen microbiome of dairy cows
Source: Transl Anim Sci. 2023 Jul 1;7(1):txad074. doi: 10.1093/tas/txad074 (PMC10362848; doi:10.1093/tas/txad074)
Supplement: txad074_suppl_Supplementary_Figures [file txad074_suppl_supplementary_figures.docx]

**Influence of dietary oils rich in omega-6 or omega-3 fatty acids on rumen microbiome of dairy cows**

Nathaly Cancino-Padilla^1,2^, Felipe Gajardo^3^, André Luis Alves Neves^4^, Ahmed Eid Kholif^5^, Marcello Mele^6^, Sharon A. Huws^7^, Juan J. Loor^8^, Jaime Romero^3,*^, Einar Vargas-Bello-Pérez^9, 1*^

^1^ Pontificia Universidad Católica de Chile, Departamento de Ciencias Animales, Facultad de Agronomía e Ingeniería Forestal, Avda Vicuña Mackenna 4860, Santiago, Chile.

^2^ Instituto de Investigaciones Agropecuarias, INIA Carillanca, Temuco, Chile.

^3^ Universidad de Chile, Instituto de Nutrición y Tecnología de los Alimentos (INTA), Av. El Líbano 5524, Macul, Santiago, Chile

^4^ Production, Nutrition and Healt h, Department of Veterinary and Animal Sciences, University of Co-penhagen, Grønnegårdsvej 3, 1870 Frederiksberg C, Denmark

^5^ Dairy Science Department, National Research Centre, 33 Bohouth St. Dokki, Giza, Egypt

^6^ Dipartimento di Scienze Agrarie, Alimentari e Agro-ambientali, Università di Pisa, Via del Borghetto 80, 56124 Pisa, Italy

^7^ Queen’s University of Belfast, Institute for Global Food Security, School of Biological Sciences, 97 Lisburn Road Belfast, BT9 7BL, United Kingdom.

^8^ Department of Animal Sciences and Division of Nutritional Sciences, University of Illinois, Mammalian NutriPhysioGenomics, Urbana 61801, USA

^9^ Department of Animal Sciences, School of Agriculture, Policy and Development, University of Reading, P.O. Box 237, Earley Gate, Reading RG6 6EU, U.K.

Correspondence: [e.vargasbelloperez@reading.ac.uk](mailto:e.vargasbelloperez@reading.ac.uk) and [jromero@inta.uchile.cl](mailto:jromero@inta.uchile.cl)

**Supplementary Material**


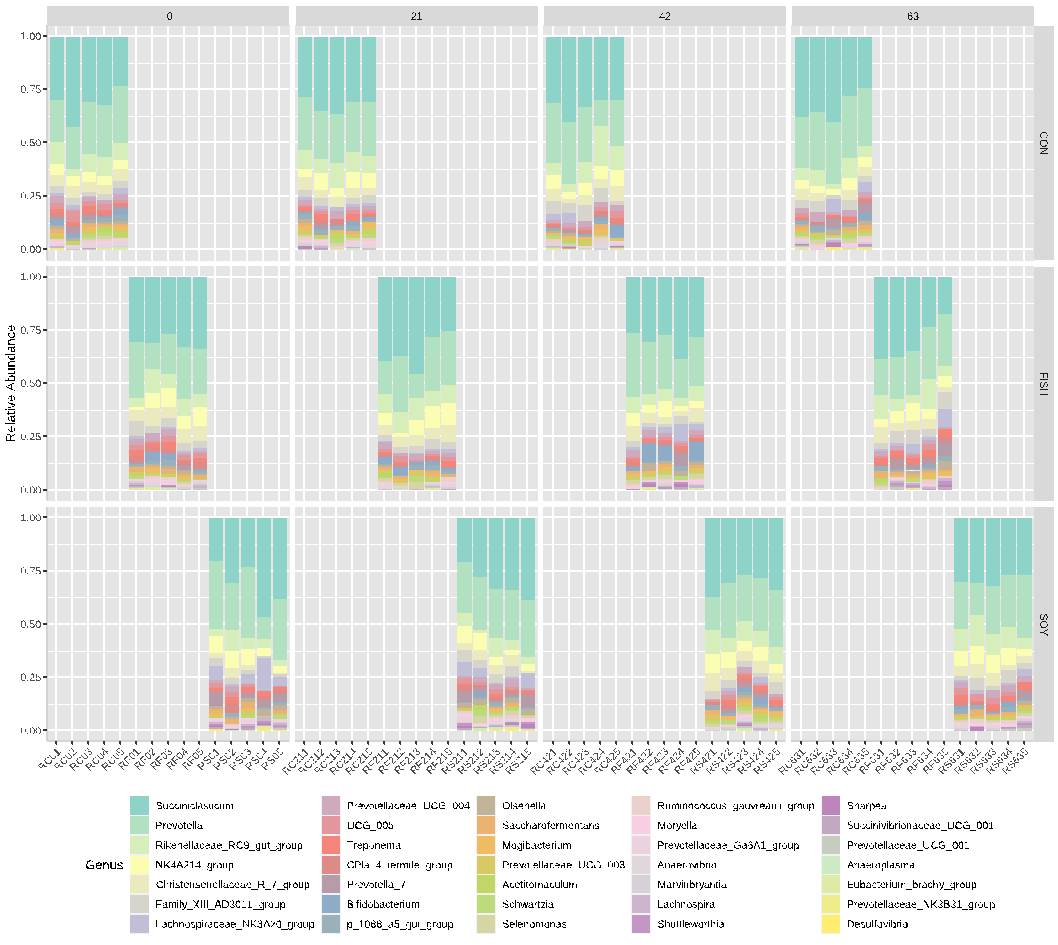


**Figure S1.** Microbiota composition at genus level. Microbiota during the assay at days 0, 21, 42 and 63 and cows fed different diets (control, soybean oil and fish oil). Relative abundance is showed in the scale 0 to 1.


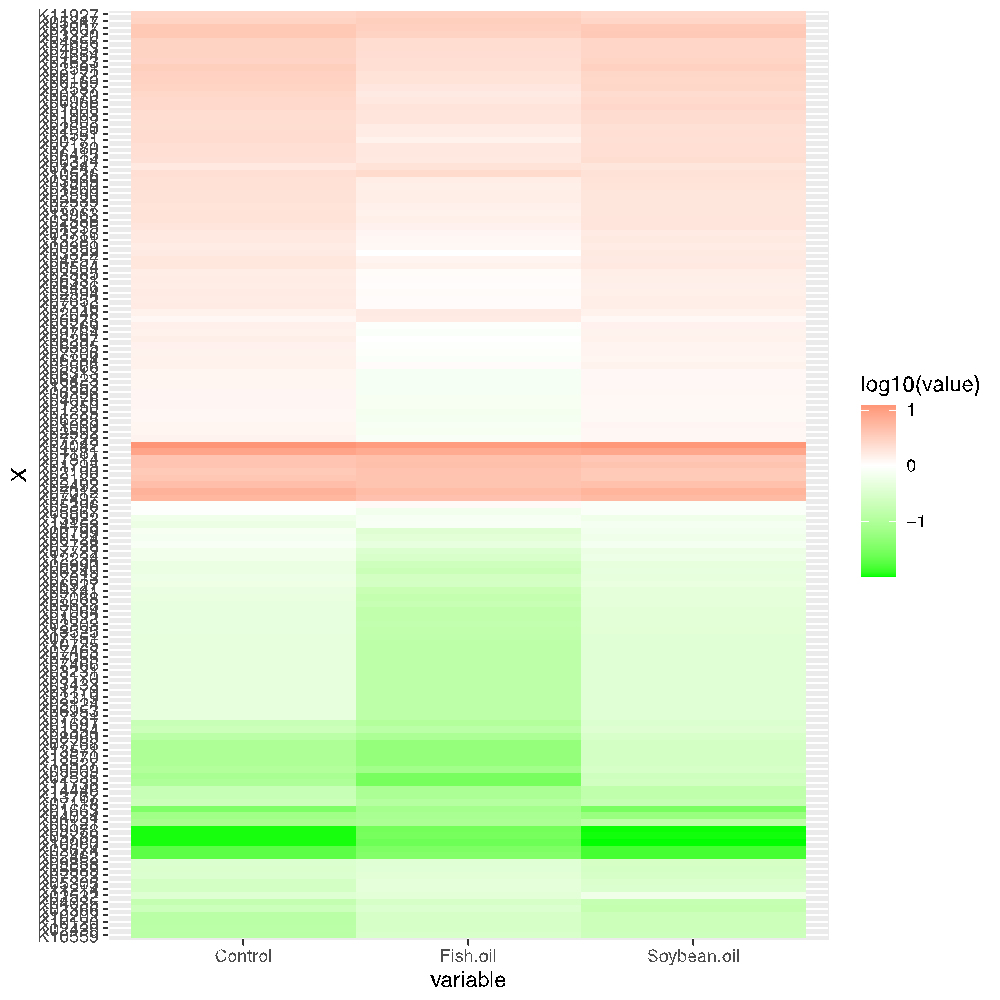


**Figure S2.** Heatmap of 140 metabolic pathways distributed differentially between control, soybean oil and fish oil (lefse).
